# Trajectory of Health-Related Quality of Life After Pediatric Epilepsy Surgery

**DOI:** 10.1001/jamanetworkopen.2023.4858

**Published:** 2023-03-27

**Authors:** Elysa Widjaja, Klajdi Puka, Kathy N. Speechley, Mark A. Ferro, Mary B. Connolly, Philippe Major, Anne Gallagher, Salah Almubarak, Simona Hasal, Rajesh Ramachandrannair, Andrea Andrade, Qi Xu, Edward Leung, O. Carter Snead, Mary Lou Smith

**Affiliations:** 1 Department of Diagnostic Imaging, The Hospital for Sick Children, Toronto, Ontario, Canada; 2 Division of Neurology, The Hospital for Sick Children, Toronto, Ontario, Canada; 3 Department of Medical Imaging, Lurie Children’s Hospital, Chicago, Illinois; 4 Institute for Mental Health Policy Research, Centre for Addiction and Mental Health, Toronto, Ontario, Canada; 5 Department of Epidemiology and Biostatistics, Western University, London, Ontario, Canada; 6 Department of Paediatrics, Schulich School of Medicine and Dentistry, Western University, London, Ontario, Canada; 7 School of Public Health Sciences, University of Waterloo, Waterloo, Ontario, Canada; 8 Division of Neurology, Department of Pediatrics, BC Children’s Hospital, Vancouver, British Columbia, Canada; 9 Division of Neurology, Department of Pediatrics, CHU Ste-Justine Hospital, University of Montreal, Quebec, Canada; 10 Centre de Recherche, CHU Ste-Justine Hospital, University of Montreal, Quebec, Canada; 11 Division of Pediatric Neurology, Department of Pediatrics, University of Saskatchewan, Saskatoon, Saskatchewan, Canada; 12 Qatif Central Hospital, Qatif, Saudi Arabia; 13 Department of Pediatrics, McMaster University, Hamilton, Ontario, Canada; 14 Department of Pediatrics and Child Health, University of Manitoba, Winnipeg, Manitoba, Canada; 15 Department of Psychology, University of Toronto Mississauga, Mississauga, Ontario, Canada

## Abstract

**Importance:**

Health-related quality of life (HRQOL) is regarded as a key outcome for evaluating treatment efficacy. However, it is uncertain how HRQOL evolves after epilepsy surgery compared with medical therapy, such as whether it continues to improve over time, improves and then remains stable, or deteriorates after a period of time.

**Objective:**

To assess trajectory of HRQOL over 2 years in children with drug-resistant epilepsy (DRE) treated with surgery compared with medical therapy.

**Design, Setting, and Participants:**

Prospective cohort study assessing HRQOL longitudinally over 2 years. Participants were children recruited from 8 epilepsy centers in Canada from 2014 to 2019 with suspected DRE aged 4 to 18 years who were evaluated for surgery. Data were analyzed from May 2014 to December 2021.

**Exposures:**

Epilepsy surgery or medical therapy.

**Main Outcomes and Measures:**

HRQOL was measured using the Quality of Life in Childhood Epilepsy Questionnaire (QOLCE)-55. HRQOL and seizure frequency were assessed at baseline, 6-month, 1-year, and 2-year follow-ups. Clinical, parent, and family characteristics were assessed at baseline. A linear mixed model was used to evaluate HRQOL over time, adjusting for baseline clinical, parent, and family characteristics.

**Results:**

There were 111 surgical and 154 medical patients (mean [SD] age at baseline was 11.0 [4.1] years; 118 [45%] were female). At baseline, HRQOL was similar among surgical and medical patients. HRQOL of surgical patients was 3.0 (95% CI, −0.7 to 6.8) points higher at 6-month, 4.9 (95% CI, 0.7 to 9.1) points higher at 1-year, and 5.1 (95% CI, 0.7 to 9.5) points higher at 2-year follow-ups compared with medical patients. Surgical patients experienced greater improvements in social functioning relative to medical patients, but not for cognitive, emotional, and physical functioning. At 2-year follow-up, 72% of surgical patients were seizure-free, compared with 33% of medical patients. Seizure-free patients reported higher HRQOL than those who were not.

**Conclusions and Relevance:**

This study provided evidence on the association between epilepsy surgery and children’s HRQOL, with improvement in HRQOL occurring within the first year and remaining stable 2 years after surgery. By demonstrating that surgery improved seizure freedom and HRQOL, which has downstream effects such as better educational attainment, reduced health care resource utilization, and health care cost, these findings suggest that the high costs of surgery are justified, and that improved access to epilepsy surgery is necessary.

## Key Points

**Question:**

In children with drug-resistant epilepsy, how did the trajectory of health-related quality of life (HRQOL) change over 2 years after surgery compared with medical therapy?

**Findings:**

This multicenter cohort study of 265 patients showed that among surgical patients, improvement in HRQOL occurred in the first year after surgery, with the largest improvement occurring in the first 6 months after surgery, and thereafter remained stable at 24 months after surgery. Among medical patients, HRQOL remained unchanged over the 2-year follow-up.

**Meaning:**

This study provided evidence on the association between epilepsy surgery and children’s HRQOL, as well as the evolution of HRQOL after surgery.

## Teaser

This cohort study evaluates the trajectory of health-related quality of life after pediatric epilepsy surgery.

## Introduction

Epilepsy affects 0.5% to 1% of children^1^ and can have negative consequences on multiple facets of children’s health-related quality of life (HRQOL), including their behavior, cognitive, social, and emotional functioning.^2,3^ Drug-resistant epilepsy (DRE) refers to continuation of seizures despite adequate trials of 2 or more antiseizure medications (ASMs), and affects 25% to 40% of those with epilepsy.^4,5^ Epilepsy surgery has been shown to improve seizure control in children with DRE^6^ and is the recommended treatment for focal DRE. Because epilepsy affects multiple facets of the patients’ lives, multidimensional HRQOL is a highly appropriate patient-centered outcome for evaluating the effectiveness of epilepsy surgery. HRQOL has been regarded by the International League Against Epilepsy Commission on Epidemiology as a key measurement for evaluating treatment efficacy.^7^

A prior meta-analysis based on retrospective studies showed that epilepsy surgery in children was associated with better HRQOL compared with presurgery and with medical therapy.^8^ Furthermore, children who were seizure-free after surgery demonstrated significant improvement in HRQOL, while those who continued to have seizures did not. However, there is lack of consensus on which aspects of HRQOL improve with seizure freedom after surgery^9,10,11,12^ and when it improves. Previous studies showed that improvement in social function and social problem subscales took time to emerge after epilepsy surgery, with no changes in these subscales in the first year after surgery,^13^ and improvements were observed only in the second year after surgery.^14^ Most published studies are retrospective studies, lack a control group for comparison,^9,10,11,12^ are cross-sectional,^10,12,15,16,17^ or assessed HRQOL at only 2 time points.^9,18^ Hence, it is also unclear how HRQOL evolves after surgery compared with medical therapy, whether it continues to improve over time, improves and then remains stable, or deteriorates after a period of time. This study addressed these knowledge gaps by using a multicenter prospective cohort design with a large sample size, including a medical therapy group for comparison, and measuring outcomes longitudinally to assess trajectories of HRQOL over time in the 2 treatment groups. Furthermore, we considered a wide range of clinical, parent, and family characteristics, as these factors have been shown to be associated with children’s HRQOL.^19,20,21^

The aim of the study was to assess trajectory of HRQOL over 2 years in children with DRE treated with surgery compared with medical therapy. The hypothesis was that epilepsy surgery would result in greater improvement in HRQOL in children with DRE compared with medical therapy, and the improvement would be evident within the first year after surgery and maintained in the second year after surgery.

## Methods

### Study Design and Setting

This multicenter prospective cohort study recruited children aged 4 to 18 years with DRE treated with epilepsy surgery or medical therapy from 8 centers in Canada from 2014 to 2019, including the Hospital for Sick Children in Toronto, British Columbia Children’s Hospital in Vancouver, Alberta Children’s Hospital in Calgary, Centre Hospitalier Universitaire Sainte-Justine in Montreal, The Children’s Hospital in Winnipeg, Royal University Hospital in Saskatoon, McMaster Children’s Hospital in Hamilton, and London Health Sciences Centre in London. The study protocol was approved by the research ethics boards of the participating hospitals, and written informed consent and assent was obtained from parents and children with DRE. We followed the Strengthening the Reporting of Observational Studies in Epidemiology (STROBE) reporting guideline.^22^ Data were collected and managed using the Research Electronic Data Capture (REDCap).^23^

### Participants

All children had suspected focal epilepsy as assessed by clinical semiology and/or electroencephalography (EEG) and were being evaluated for epilepsy surgery candidacy. Exclusion criteria included previous epilepsy surgery, prior or planned palliative surgery (eg, corpus callosotomy or vagus nerve stimulator placement), neurometabolic disorders, neurodegenerative disorders, genetic epilepsy syndromes and epileptic encephalopathies not amenable to surgery. All patients underwent detailed clinical assessment, magnetic resonance imaging, video EEG, and neuropsychological testing, supplemented by additional neurodiagnostic tests as deemed necessary for epilepsy surgery evaluation. The decision to exclude a child from epilepsy surgery was made as part of clinical care, for reasons such as inability to localize or lateralize a single epileptogenic focus, and anticipated neurological deficits if surgery were to proceed. The medical group consisted of children who, after undergoing a standard evaluation for epilepsy surgery candidacy, were deemed ineligible for surgery. The outcomes of a subgroup of these patients have been reported in prior publications.^24,25^ Participants who completed the baseline assessment only were excluded from analysis.

### HRQOL Outcome

HRQOL was measured using the parent-reported Quality of Life in Childhood Epilepsy Questionnaire (QOLCE)-55. It assesses 4 domains of HRQOL: physical, cognitive, emotional, and social function. The total QOLCE-55 score is the unweighted mean of the 4 subscales, with higher score indicating better HRQOL. The QOLCE-55 has demonstrated good reliability and validity,^26,27^ including in children with DRE^28^ and young adults with childhood-onset epilepsy.^29^ In this study, the internal consistency of the QOLCE-55 ranged from α = .96 to .97. HRQOL was assessed at baseline (time of surgical evaluation), and at 6-month, 1-year, and 2-year follow-ups.

### Clinical Characteristics

The clinical data at baseline including age, sex, age at seizure onset, side and site of seizure focus, and number of ASMs were obtained from medical records. Parents were asked to report on their child’s seizure frequency at baseline and the 6-month, 1-year, and 2-year follow-ups.

### Parent and Family Characteristics

Parent characteristics at baseline included age, sex, employment status, household income, education, and marital status. Three measures of family functioning were completed by parents at baseline. The Family Adaptability, Partnership, Growth, Affective, and Resolve (Family APGAR)^30,31^ scale assessed satisfaction with family relationships. Family resources were examined using the family mastery and health, and extended family social support subscales from the Family Inventory of Resources for Management,^32^ which measures the availability of family resources to assist family adaptation for stressful life events. Family demands were evaluated using the Family Inventory of Life Events and Changes,^33^ which assesses normal and nonnormal life events experienced by the family. The reliability and validity of all 3 measures of family functioning have previously been established.^31,32,33^

### Statistical Analysis

To compare the means and proportions of baseline clinical variables and QOLCE-55 for surgery and medical groups, *t* tests or χ^2^ tests were used, respectively. A linear mixed model was used to evaluate total HRQOL over time,^34^ using random intercepts and slopes for children over time, and fixed effects for years of follow-up (continuous), group (surgery vs medical), and their interaction, as well as baseline variables previously shown to be associated with children’s HRQOL. These baseline variables include clinical (age of onset, number of ASMs, and seizure frequency), parent and family characteristics (parent education, parent marital status, family resources, family relationships, and family demands).^19,20,21^ Years of follow-up was calculated as the years since baseline. The likelihood ratio test showed that the inclusion of random slopes and a quadratic term for years of follow-up significantly improved model fit. Study site was not included in the model as a random factor given that its variance was estimated to be 0. The linear mixed model used restricted maximum likelihood estimation (REML), a Gaussian distribution, and Satterthwaite method to approximate the degrees of freedom.^35^ The emmeans package in R was used to decompose interactions.^36^ Model assumptions were checked, and no violations were identified. The linear mixed model included all participants in the analyses by using REML to accommodate missing HRQOL data over time, whereas missing data on baseline covariates (5 participants [1%]) were handled using complete case analyses. All analyses were completed using R version 4.2.0 (R Project for Statistical Computing). Data were analyzed from May 2014 to December 2021.

To evaluate change at the individual level, the proportions of surgical and medical patients who showed meaningful improvement, meaningful deterioration, or stable HRQOL scores from baseline to the 2-year follow-up were compared using χ^2^ tests. Meaningful change was based on a change of more than 7.25 points; this minimal clinically important difference (MCID) value was derived from a population-based study of children with epilepsy,^37^ and calculated as half a standard deviation (SD) of the total QOLCE-55 score.^38^

#### Sensitivity Analyses

The linear mixed model described previously was repeated for each QOLCE subscale. We also compared the proportion of patients who showed meaningful improvement, meaningful deterioration, or stable QOLCE subscale scores from baseline to 2 years for the 2 groups using χ^2^ tests. The linear mixed model was repeated for HRQOL, with seizure status (seizure-free vs not seizure-free) included as a time-varying covariate, as well as its 3-way interaction with years of follow-up and group.

#### Sample Size

The sample size calculation used data from a population-based study of children with epilepsy.^37^ The mean (SD) total QOLCE-55 at the 2-year follow-up was 76.4 (14.5) and the MCID was 7.25. The correlation of scores among participants over time (intraclass correlation coefficient) was 0.73. Using these estimates, a simulation study using the simr package,^39^ with 4 measurement occasions, and 20% missing data suggested that a linear mixed model with 60 participants per group will have 90% statistical power to detect the MCID in HRQOL between surgical- and medical-treated children at α = .05. Speechley et al^37^ also identified that the intraclass correlation coefficient associated with recruiting participants from the same study site was 0.005, resulting in a design effect of 1.17. Accounting for this clustering effect, the minimum required sample size per group is 70 participants.

## Results

### Participants

**Figure 1.**
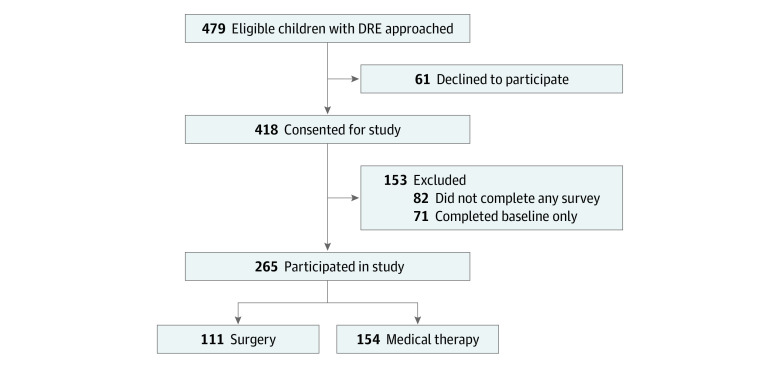
Flowchart Showing the Number of Eligible Children With Drug-Resistant Epilepsy (DRE) and Those Who Participated in the Study

Of the 479 children eligible for the study, 265 participated (Figure 1). Their mean (SD) age at baseline was 11.0 (4.1) years, 118 (45%) were female, 148 (56%) had daily or weekly seizures, and they were receiving a mean (SD) of 2.0 (0.9) ASM (Table 1). Among these 265 children, 170 were followed over 2 years; all children with missing data at follow-ups were included. Children who completed the 2-year follow-up had similar baseline characteristics to those who did not (eTables 1 and 2 in Supplement 1), with the exception of better family resources and HRQOL among children who completed the 2-year follow-up. The proportion of surgical and medical patients followed over 2 years was not statistically different (χ^2^_1_ = 1.82; *P* = .17).

**Table 1.** Baseline Demographic and Clinical Characteristics

Child characteristics	No. (%)			*P* value
	Overall (N = 265)	Medical (n = 154)	Surgery (n = 111)	
Sex				
Female	118 (44.5)	73 (47.4)	45 (40.5)	.33
Male	147 (55.5)	81 (52.6	66 (59.5)	
Age, mean (SD), y				
At seizure onset	6.3 (4.1)	6.0 (4.1)	6.8 (4.1)	.12
At baseline	11.0 (4.1)	10.6 (4.0)	11.5 (4.2)	.11
Side of seizure focus				
Right	98 (37.0)	50 (32.5)	48 (43.2)	<.001
Left	129 (48.7)	67 (43.5)	62 (55.9)	
Bilateral	38 (14.3)	37 (24.0)	1 (0.9)	
Site of seizure focus				
Temporal	78 (29.4)	32 (20.8)	46 (41.4)	<.001
Frontal	75 (28.3)	42 (27.3)	33 (29.7)	
Parietal	16 (6.0)	4 (2.6)	12 (10.8)	
Occipital	13 (4.9)	10 (6.5)	3 (2.7)	
Multilobar	58 (21.9)	41 (26.6)	17 (15.3)	
Unknown	25 (9.4)	25 (16.2)	0 (0.0)	
Seizure frequency				
Daily	65 (24.5)	30 (19.5)	35 (31.5)	.02
Weekly	83 (31.3)	47 (30.5)	36 (32.4)	
Monthly	59 (22.3)	35 (22.7)	24 (21.6)	
Yearly	45 (17.0)	30 (19.5)	15 (13.5)	
No seizures in the last year	13 (4.9)	12 (7.8)	1 (0.9)	
No. of antiseizure medications, mean (SD)	2.0 (0.9)	2.0 (1.0)	1.9 (0.7)	.47
QOLCE-55 total score, mean (SD)	57.6 (17.7)	57.2 (18.0)	58.2 (17.4)	.66
Parent and family characteristics				
Sex				
Female	222 (83.8)	134 (87.0)	88 (79.3)	.13
Male	43 (16.2)	20 (13.0)	23 (20.7)	
Age				
<30 y	10 (3.8)	7 (4.5)	3 (2.7)	.02
30-39 y	88 (33.2)	62 (40.3)	26 (23.4)	
40-49 y	133 (50.2)	69 (44.8)	64 (57.7)	
≥50 y	34 (12.8)	16 (10.4)	18 (16.2)	
Working or a student	203 (76.6)	117 (76.0)	86 (77.5)	.89
College/university or more	184 (69.4)	108 (70.1)	76 (68.5)	.88
Married or living with spouse	224 (84.5)	127 (82.5)	97 (87.4)	.36
Household income				
<$50 000	42 (16.5)	25 (17.2)	17 (15.6)	.42
$50 000-$99 999	101 (39.8)	63 (43.4)	38 (34.9)	
$100 000-$149 999	55 (21.7)	29 (20.0)	26 (23.9)	
≥$150 000	56 (22.0)	28 (19.3)	28 (25.7)	
Family relationships, APGAR, mean (SD)	7.2 (2.3)	7.4 (2.2)	6.9 (2.5)	.09
Family resources, FIRM, mean (SD)	49.8 (11.1)	49.7 (11.0)	49.8 (11.3)	.95
Family demands, FILE, mean (SD)	9.3 (6.3)	9.9 (6.4)	8.4 (5.9)	.05

Abbreviations: APGAR, Family Adaptability, Partnership, Growth, Affective, and Resolve scale; FILE, Family Inventory of Life Events and Changes; FIRM, Family Inventory of Resources for Management; QOLCE, Quality of Life in Childhood Epilepsy Questionnaire.

There were 111 surgical and 154 medical patients. Surgical patients were more likely to have more frequent seizures, unilateral temporal seizures, or extratemporal seizures, while medical patients were more likely to have bilateral, multilobar, or unknown seizure focus. Parent and family characteristics of surgical and medical patients were similar, with the exception of parental age, with more parents 40 years and older in the surgical group and more parents younger than 39 years in the medical group.

### HRQOL of Surgical and Medical Patients

**Figure 2.**
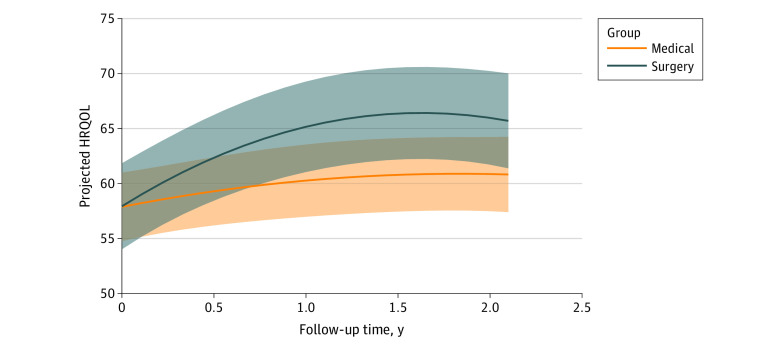
Model Projected Health-Related Quality of Life (HRQOL) Trajectories

**Figure 3.**
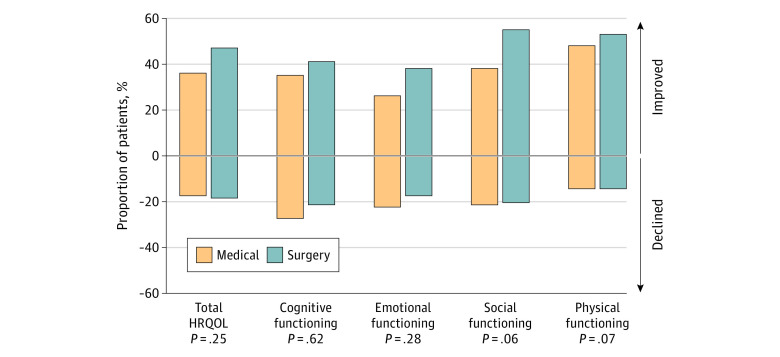
Proportion of Patients Who Showed Improved (+7.25 Points) and Declined (−7.25 Points) Health-Related Quality of Life Scores at the 2-Year Follow-up, Relative to Baseline The listed *P* values present the results of χ^2^ analyses comparing the proportion of surgical and medical patients who showed improved, declined, and unchanged scores.

HRQOL (total QOLCE-55) scores across the 2-year follow-up are shown in Table 2. The linear mixed model showed a significant group by time interaction indicating that surgical patients experienced significantly greater HRQOL improvement relative to medical patients, while adjusting for multiple baseline clinical, parent, and family characteristics (Figure 2; eTable 3 in Supplement 1). Decomposing this interaction showed that HRQOL was similar among surgical and medical patients at baseline (mean difference, 0.0; 95% CI, −3.8 to 3.9). Relative to medical patients, the HRQOL of surgical patients was a mean of 3.0 (95% CI, −0.7 to 6.8) points higher at 6 months, 4.9 (95% CI, 0.7 to 9.1) points higher at 1 year, and 5.1 (95% CI, 0.7 to 9.5) points higher at 2 years. The model also showed that higher HRQOL was associated with older age of seizure onset, fewer ASMs, and greater family resources at baseline. There were no significant differences in the proportion of surgical and medical patients who showed a meaningful change in HRQOL at the individual level (χ^2^_2_ = 2.76*; P* = .25) (Figure 3).

**Table 2.** Quality of Life in Childhood Epilepsy Questionnaire-55 Total and Subscale Scores at Each Follow-up

Group	Time	No.	Mean (SD)					
			Follow-up time, y^a^	Total	Cognitive	Emotional	Social	Physical
Medical	Baseline	154	NA	57.21 (18.00)	52.46 (23.34)	67.30 (15.05)	62.38 (27.69)	46.70 (21.73)
	6 mo	129	0.52 (0.1)	59.58 (19.18)	53.88 (24.83)	69.11 (15.42)	65.12 (27.98)	50.24 (23.65)
	1 y	132	1.07 (0.20)	59.76 (19.02)	50.62 (24.40)	69.53 (14.96)	66.50 (27.92)	52.65 (24.06)
	2 y	104	2.10 (0.23)	62.15 (19.09)	55.63 (25.70)	69.15 (14.68)	70.15 (27.23)	54.01 (25.43)
Surgery	Baseline	111	NA	58.18 (17.42)	55.13 (22.94)	66.79 (15.13)	60.75 (27.47)	50.08 (21.07)
	6 mo	84	0.51 (0.13)	63.83 (18.16)	58.47 (22.28)	71.48 (14.60)	70.66 (27.64)	54.70 (23.70)
	1 y	94	1.11 (0.20)	65.91 (17.07)	59.82 (21.69)	70.06 (14.90)	76.90 (25.76)	56.86 (22.41)
	2 y	66	2.13 (0.24)	67.43 (18.17)	61.76 (23.22)	71.37 (14.57)	77.76 (26.69)	58.84 (22.23)

Abbreviation: NA, not applicable.

^a^ Mean years since baseline assessment (medical patients) or surgery (surgical patients).

### Sensitivity Analyses

Surgical patients experienced significantly greater improvements in the social functioning subscale relative to medical patients (eFigure 1 in Supplement 1). Improvement in social functioning occurred 6 months and 1 year after surgery and remained stable 2 years after surgery (Table 2). There were no significant differences in cognitive, emotional, and physical functioning among surgical and medical patients (eTable 4 in Supplement 1).

At the 2-year follow-up, 72% of surgical patients were seizure-free, compared with 33% of medical patients (eTable 5 in Supplement 1). There was a significant time by group by seizure status interaction (eTable 6 in Supplement 1). Patients who were seizure-free reported significantly higher HRQOL than those who were not seizure-free throughout the follow-up, irrespective of surgical status (eFigure 2 in Supplement 1).

## Discussion

To our knowledge, this was the largest prospective observational study assessing HRQOL after epilepsy surgery in children, and the inclusion of surgical and medical patients from multiple centers enhanced the generalizability of study findings. We observed that surgery was associated with higher HRQOL compared with medical therapy in children with DRE. The longitudinal design allowed us to evaluate how HRQOL evolved over time following the 2 treatments. We found that the largest improvement in HRQOL among surgical patients occurred in the first 6 months after surgery. However, HRQOL continued to improve in the first year after surgery, and thereafter remained stable 2 years after surgery. Among medical patients, HRQOL remained relatively unchanged over the 2-year follow-up. Social functioning was the only subscale that showed improvement after surgery, whereas cognitive, emotional, and physical functioning subscales did not improve after surgery. Higher seizure freedom following surgery could have been associated with the higher HRQOL among surgical compared with medical patients.

The evolution of HRQOL over time after epilepsy surgery is uncertain, as most studies assessing HRQOL after surgery are cross-sectional,^10,12,15,16,17^ and few studies measured HRQOL before and after surgery across 2 time points.^9,18^ Van Empelen et al^40^ evaluated the HRQOL of 21 children after epilepsy surgery and showed that improvement in the frequency and/or quality of their physical and social activities as well as positive emotions were evident 6 months after surgery, and remained stable 12 and 24 months after surgery. The authors reported HRQOL subscales but did not provide an estimate of overall HRQOL over time. Furthermore, they compared outcomes at each time point relative to the most recent prior time point (eg, 12-month compared with 6-month assessment), rather than evaluating the trajectory of HRQL over time. The present study contributes to the literature by using a longitudinal design and growth curve analysis to examine the trajectory of HRQOL over 2 years after pediatric epilepsy surgery.

Improvement in HRQOL among surgical patients was mainly associated with the increase in social functioning. The largest increase in social functioning occurred in the first 6 months after surgery but the increase continued in the next 6 months and then remained fairly stable 2 years after surgery. Van Empelen et al^40^ found that improvement in social functioning occurred in the first 6 months after surgery and then remained stable from 6 to 24 months after surgery. Other studies found no change in social function 1 year after surgery,^13^ but significant improvements among surgical patients occurred 2 years after surgery.^14^ Our prior study^24^ showed that surgery had a direct and indirect effect on improving social functioning, and the indirect effect was mediated by seizure freedom. A qualitative study found that children who were seizure-free after surgery reported increased satisfaction with their peer relationships and experienced greater social inclusion.^41^ Seizure freedom after surgery improved patients’ autonomy, reduced fear and anxiety related to the unpredictability of seizures, improved patients’ confidence, and enhanced opportunities for social interactions.^41^ This study did not show improvement in physical, cognitive, or emotional functioning after surgery compared with medical therapy. Our prior study also demonstrated that surgery did not have a direct effect on physical or cognitive functioning, but had an indirect effect on these functions that were mediated by seizure freedom.^24^

Although we have assessed the evolution of HRQL over 2 years, the longer-term trajectory of HRQOL remains to be evaluated. Previous studies have found that the long-term HRQOL in young adults who have epilepsy surgery in childhood or who continued on medical therapy was dependent upon seizure status rather than treatment group.^42,43^ Among surgical patients who were seizure-free, length of follow-up between 4 and 11 years did not influence HRQOL.^43^ Seizure freedom after surgery may not be sustained^44,45,46,47^ as the brain may develop a new epileptogenic area.^48^ Seizure freedom at 5 or more years after surgery has been estimated to worsen by about 20% compared with 1 or 2 years after surgery.^44,45,46,47^ Given the potential decline in seizure freedom over time after surgery, it is uncertain whether improvement in HRQOL is sustained in the long-term.

### Limitations

There are limitations to this study. Although the ideal study design would have been a randomized clinical trial, we anticipate that there would be challenges in recruiting children for such a trial as epilepsy surgery is the standard-of-care treatment for children with focal DRE who are suitable candidates for surgery. It would be ethically unacceptable to withhold surgical treatment from children who are considered suitable candidates for surgery. Given the previously stated considerations, we conducted a prospective observational study. To our knowledge, this is the largest multicenter study evaluating the trajectory of HRQOL following pediatric epilepsy surgery compared with medical therapy. Although there were differences in a few clinical characteristics of surgical and medical patients at baseline, we have comprehensively adjusted for those variables that have been reported to influence HRQOL in the analysis. Furthermore, the 2 groups did not differ in their baseline HRQOL, which is one of the most important predictors of HRQOL at follow-up.^9,24^ Another limitation is the use of parent-proxy-report rather than child report of the child’s HRQOL. In general, parents tend to underestimate their child’s HRQOL, with greater disagreement between child- and parent-reports across less observable domains (such as emotional functioning) relative to observable domains (such as physical functioning).^49,50^ Van Empelen et al^40^ found that children rated their social, physical and cognitive functioning higher compared with parents after epilepsy surgery. General agreement in the trajectories of child- vs parent-report of HRQOL in children with epilepsy have been reported,^51^ supporting previous evidence that parents are valid proxy informants. However, slight differences in trajectories were detected, suggesting that multi-informant approaches are important to provide a broader view of child HRQOL over time.

## Conclusions

This multicenter prospective observational study provided evidence on the association between epilepsy surgery and children’s HRQOL. Higher seizure freedom following epilepsy surgery could have been associated with improvement in HRQOL. Understanding the evolution of HRQOL after surgery compared with medical therapy is important in counseling children and parents and will contribute to informed decision-making on treatment options. By demonstrating that epilepsy surgery leads to improvement in seizure freedom and HRQOL in children, which has downstream effects such as better educational and vocational attainment and reduced health care resource utilization^52,53^ and health care cost,^54^ our study provided objective evidence to justify the high costs of surgery and to improve access to surgery.

## Notes

Supplement 1. **eTable 1. **Comparison of Baseline Demographic and Clinical Characteristics Among Participants Who Did and Did Not Complete the 2-Year Follow-up **eTable 2. **QOLCE-55 Total Score Among Those Who Completed 2-Year Follow-up vs Those Who Did Not **eTable 3. **Results of the Linear Mixed Model Evaluating Health-Related Quality of Life (HRQOL) Over Time for Surgical and Medical Patients, While Adjusting for Baseline Characteristics **eFigure 1. **Model Predicted Health-Related Quality of Life (HRQOL) Trajectories for Each Subscale **eTable 4. **Results of Linear Mixed Models for Each Health-Related Quality of Life (HRQOL) Subscale **eTable 5. **Comparison of Patients With and Without Seizures at Each Time Point, Stratified by Group **eTable 6. **Results of Linear Mixed Model Evaluating a 3-Way Interaction of Time, Surgical Status and Seizure Status **eFigure 2. **Model Predicted Health-Related Quality of Life (HRQOL; Total QOLCE-55 Score) Trajectories, Focused on the 3-Way Interaction Between Time, Surgical Status, and Seizure Status

Supplement 2.
Data Sharing Statement
