# Supplementary material for: Trajectory of Health-Related Quality of Life After Pediatric Epilepsy Surgery
Source: JAMA Netw Open. 2023 Mar 27;6(3):e234858. doi: 10.1001/jamanetworkopen.2023.4858 (PMC10043749; doi:10.1001/jamanetworkopen.2023.4858)
Supplement: Supplement 1. — eTable 1. Comparison of Baseline Demographic and Clinical Characteristics Among Participants Who Did and Did Not Complete the 2-Year Follow-up eTable 2. QOLCE-55 Total Score Among Those Who Completed 2-Year Follow-up vs Those Who Did Not eTable 3. Results of the Linear Mixed Model Evaluating Health-Related Quality of Life (HRQOL) Over Time for Surgical and Medical Patients, While Adjusting for Baseline Characteristics eFigure 1. Model Predicted Health-Related Quality of Life (HRQOL) Trajectories for Each Subscale eTable 4. Results of Linear Mixed Models for Each Health-Related Quality of Life (HRQOL) Subscale eTable 5. Comparison of Patients With and Without Seizures at Each Time Point, Stratified by Group eTable 6. Results of Linear Mixed Model Evaluating a 3-Way Interaction of Time, Surgical Status and Seizure Status eFigure 2. Model Predicted Health-Related Quality of Life (HRQOL; Total QOLCE-55 Score) Trajectories, Focused on the 3-Way Interaction Between Time, Surgical Status, and Seizure Status [file jamanetwopen-e234858-s001.pdf]

Supplemental Online Content

Widjaja E, Puka K, Speechley KN, et al. Trajectory of health-related quality of life after pediatric epilepsy surgery. *JAMA Netw Open*. 2023;6(3):e234858. doi:10.1001/jamanetworkopen.2023.4858

eTable 1. Comparison of Baseline Demographic and Clinical Characteristics Among Participants Who Did and Did Not Complete the 2-Year Follow-up
eTable 2. QOLCE-55 Total Score Among Those Who Completed 2-Year Follow-up vs Those Who Did Not
eTable 3. Results of the Linear Mixed Model Evaluating Health-Related Quality of Life (HRQOL) Over Time for Surgical and Medical Patients, While Adjusting for Baseline Characteristics
eFigure 1. Model Predicted Health-Related Quality of Life (HRQOL) Trajectories for Each Subscale
eTable 4. Results of Linear Mixed Models for Each Health-Related Quality of Life (HRQOL) Subscale
eTable 5. Comparison of Patients With and Without Seizures at Each Time Point, Stratified by Group
eTable 6. Results of Linear Mixed Model Evaluating a 3-Way Interaction of Time, Surgical Status and Seizure Status
eFigure 2. Model Predicted Health-Related Quality of Life (HRQOL; Total QOLCE-55 Score) Trajectories, Focused on the 3-Way Interaction Between Time, Surgical Status, and Seizure Status

This supplemental material has been provided by the authors to give readers additional information about their work.

© 2023 Widjaja E et al. *JAMA Network Open.*

eTable 1. Comparison of Baseline Demographic and Clinical Characteristics Among Participants Who Did and Did Not Complete the 2-Year Follow-up

**Child Characteristics**	Followed over 2 years (n = 170) n (%) or mean (SD)	Lost to follow-up (n= 95) n (%) or mean (SD)	p-value
Sex, female	73 (43%)	45 (47%)	.57
Age at seizure onset, years	6.3 (4.2)	6.3 (3.9)	.92
Age at baseline, years	10.9 (4.2)	11.1 (3.9)	.66
Side of seizure focus			.81
Right	63 (37%)	35 (37%)	
Left	81 (48%)	48 (51%)	
Bilateral	26 (15%)	12 (13%)	
Site of seizure focus			.19
Temporal	48 (28%)	30 (32%)	
Frontal	51 (30%)	24 (25%)	
Parietal	8 (5%)	8 (8%)	
Occipital	12 (7%)	1 (1%)	
Multi-lobar	34 (20%)	24 (25%)	
Unknown	17 (10%)	8 (8%)	
Seizure frequency			.98
Daily	40 (24%)	25 (26%)	
Weekly	53 (31%)	30 (32%)	
Monthly	38 (22%)	21 (22%)	
Yearly	30 (18%)	15 (16%)	
No seizures in the last year	9 (5%)	4 (4%)	
Number of antiseizure medications	1.9 (0.9)	2.1 (0.9)	.11
**Parent and Family Characteristics**			
Sex, male	27 (16%)	16 (17%)	.98
Age			.10
< 30 years	9 (5%)	1 (1%)	
30-39 years	61 (36%)	27 (28%)	
40-49 years	77 (45%)	56 (59%)	
≥ 50 years	23 (14%)	11 (12%)	
Working or a student	134 (79%)	69 (73%)	.32
College/University or more	124 (73%)	60 (63%)	.13
Married or living with spouse	143 (84%)	81 (85%)	.94
Household income			.13
< $50,000	22 (13%)	20 (22%)	
$50,000 - $99,999	63 (38%)	38 (42%)	
$100,000 - $149,999	41 (25%)	14 (16%)	
≥$150,000	38 (23%)	18 (20%)	
Family relationships, APGAR	7.4 (2.2)	6.9 (2.5)	.12
Family resources, FIRM	50.9 (10.9)	47.8 (11.3)	.033
Family demands, FILE	9.0 (6.1)	9.9 (6.5)	.27

© 2023 Widjaja E et al. *JAMA Network Open.*

eTable 2. QOLCE-55 Total Score Among Those Who Completed 2-Year Follow-up vs Those Who Did Not

	Completed Year 2 follow-up		Did not complete Year 2 follow-up		p-value
	n	Mean (SD)	n	Mean (SD)	
Baseline	170	59.4 (17.7)	95	54.4 (17.4)	0.027
Month 6	147	62.0 (18.4)	66	60.0 (19.7)	0.482
Year 1	150	64.5 (18.3)	72	58.1 (18.5)	0.021
Year 2	170	64.2 (18.9)	0	-	-

© 2023 Widjaja E et al. *JAMA Network Open.*

eTable 3. Results of the Linear Mixed Model Evaluating Health-Related Quality of Life (HRQOL) Over Time for Surgical and Medical Patients, While Adjusting for Baseline Characteristics

	Estimated difference (95% CI)	p-value
Years follow-up	3.3 (0.0, 6.6)	.05
Years follow-up squared	-0.9 (-2.3, 0.5)	.21
Surgery (ref = Medical)	0.0 (-3.8, 3.9)	.98
Surgery * Years follow-up	7.2 (2.0, 12.3)	.007
Surgery * Years follow-up squared	-2.3 (-4.5, -0.1)	.040
Age seizure onset	0.7 (0.2, 1.1)	.002
Number of antiseizure medications	-4.9 (-6.9, -2.8)	<.001
Seizure frequency (ref= Daily)		
Weekly	-3.0 (-7.7, 1.7)	.21
Monthly	-0.5 (-5.6, 4.7)	.85
Yearly	3.5 (-2.1, 9.1)	.22
No seizures in the last year	6.1 (-3, 15.2)	.19
Parent, college/university education	1.5 (-2.2, 5.3)	.42
Parent, married or living with spouse	3.4 (-1.5, 8.2)	.18
Family relationships, APGAR	-0.1 (-1.0, 0.7)	.79
Family resources, FIRM	0.5 (0.3, 0.8)	<.001
Family demands, FILE	-0.3 (-0.6, 0.1)	.10

© 2023 Widjaja E et al. *JAMA Network Open.*

eFigure 1. Model Predicted Health-Related Quality of Life (HRQOL) Trajectories for Each Subscale

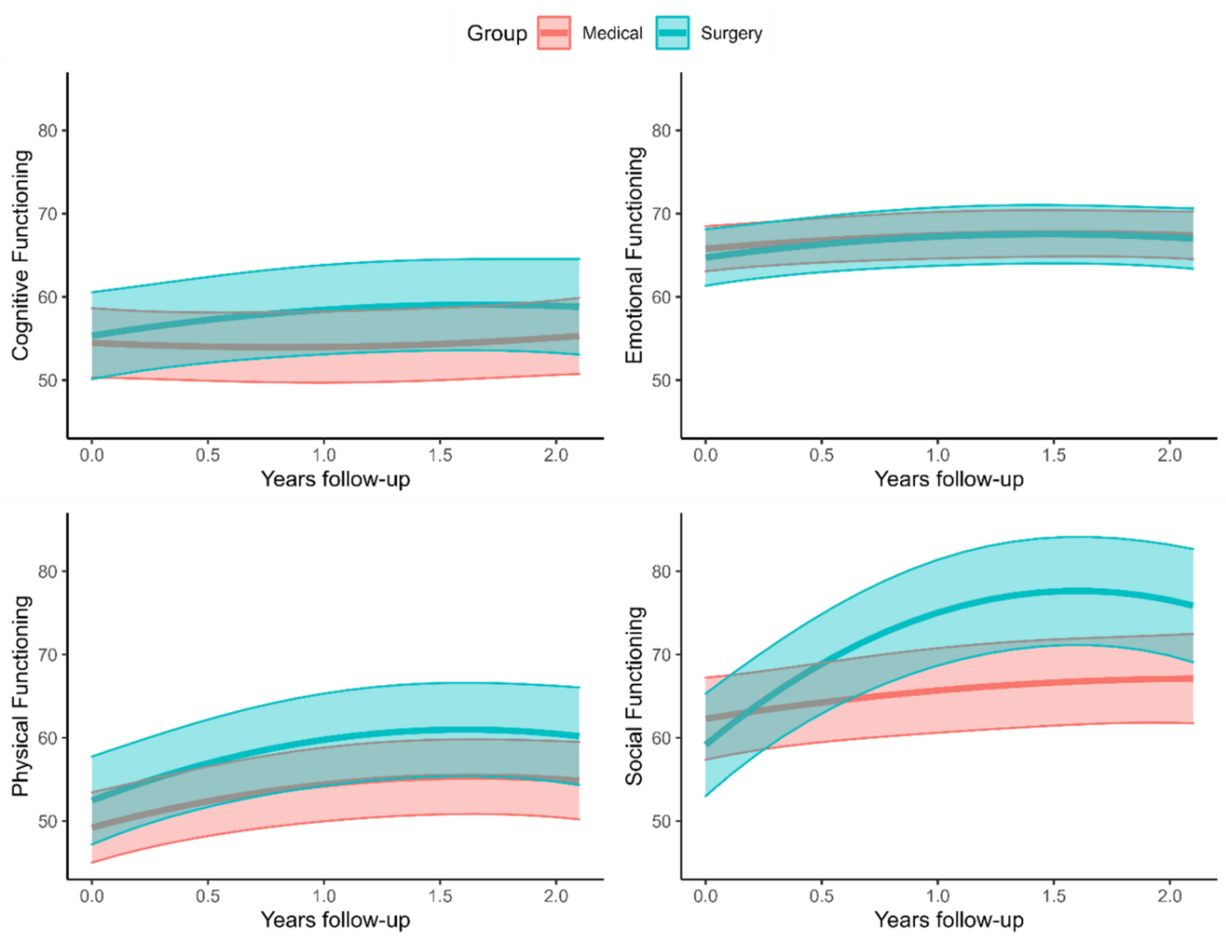

© 2023 Widjaja E et al. *JAMA Network Open.*

eTable 4. Results of Linear Mixed Models for Each Health-Related Quality of Life (HRQOL) Subscale

	Cognitive		Emotional		Social		Physical	
	Estimate (95% CI)	p-value	Estimate (95% CI)	p-value	Estimate (95% CI)	p-value	Estimate (95% CI)	p-value
Years follow-up	-1.3 (-5.4, 2.7)	.52	2.4 (-0.7, 5.4)	.12	4.4 (-1.6, 10.4)	.15	7.5 (2.8, 12.1)	.002
Years follow-up squared	0.8 (-1.0, 2.6)	.36	-0.8 (-2.1, 0.6)	.26	-1.0 (-3.5, 1.5)	.43	-2.3 (-4.3, -0.2)	.029
Surgery (ref = Medical)	0.9 (-4.3, 6.0)	.74	-1.1 (-4.4, 2.3)	.54	-3.1 (-9.3, 3.0)	.32	3.2 (-1.9, 8.4)	.22
Surgery * Years follow-up	5.8 (-0.5, 12.1)	.07	1.4 (-3.3, 6.2)	.56	18.7 (9.4, 28.0)	<.001	3.2 (-4.1, 10.4)	.39
Surgery * Years follow-up squared	-2.2 (-4.9, 0.6)	.13	-0.5 (-2.6, 1.6)	.62	-6.2 (-10.1, -2.2)	.002	-1.0 (-4.2, 2.2)	.53
Age seizure onset	1.2 (0.6, 1.8)	<.001	0.3 (-0.1, 0.6)	.14	0.2 (-0.4, 0.9)	.52	1.1 (0.5, 1.6)	<.001
Number of antiseizure medications	-5.2 (-7.9, -2.5)	<.001	-2.2 (-3.9, -0.5)	.013	-7.6 (-10.6, -4.5)	<.001	-4.8 (-7.5, -2.0)	.001
Seizure frequency (ref= Daily)								
Weekly	-5.4 (-11.7, 0.8)	.09	-3.4 (-7.4, 0.6)	.09	-2.9 (-10.1, 4.3)	.43	-0.4 (-6.8, 5.9)	.89
Monthly	0.4 (-6.5, 7.2)	.91	-3.4 (-7.8, 0.9)	.12	1.0 (-6.9, 8.9)	.81	-0.2 (-7.1, 6.8)	.96
Yearly	1.5 (-6.0, 9.0)	.69	0.1 (-4.7, 4.9)	.96	7.6 (-1.0, 16.3)	.083	3.7 (-3.9, 11.3)	.34
No seizures in the last year	5.7 (-6.5, 17.8)	.36	-2.6 (-10.3, 5.2)	.52	4.3 (-9.7, 18.2)	.55	16.5 (4.2, 28.8)	.009
Parent, college/university education	0.2 (-4.8, 5.2)	.93	0.8 (-2.4, 4.0)	.63	2.6 (-3.1, 8.4)	.37	2.3 (-2.8, 7.3)	.38
Parent, married or living with spouse	1.6 (-4.9, 8.1)	.62	6.2 (2.1, 10.4)	.003	4.7 (-2.8, 12.2)	.22	0.2 (-6.4, 6.8)	.95
Family relationships, APGAR	-1.0 (-2.1, 0.2)	.10	0.2 (-0.5, 1.0)	.52	0.1 (-1.2, 1.5)	.82	-0.1 (-1.2, 1.1)	.91
Family resources, FIRM	0.7 (0.4, 1.0)	<.001	0.4 (0.2, 0.6)	<.001	0.6 (0.3, 0.9)	<.001	0.4 (0.1, 0.7)	.003
Family demands, FILE	-0.5 (-0.9, 0.0)	.05	-0.3 (-0.6, 0.0)	.029	-0.4 (-0.9, 0.2)	.19	-0.1 (-0.5, 0.4)	.79

© 2023 Widjaja E et al. *JAMA Network Open.*

eTable 5. Comparison of Patients With and Without Seizures at Each Time Point, Stratified by Group

Group	Years Follow-up	Seizure-free, % (n)	Mean difference (95% CI) *
Surgery	0.0	1% (1)	11.8 (4.8, 18.8)
	0.5	37% (31)	7.6 (4.1, 11.1)
	1.0	65% (60)	5.1 (1.8, 8.5)
	2.0	72% (49)	5.5 (0.9, 10.1)
Medical	0.0	8% (12)	2.3 (-3.3, 7.8)
	0.5	16% (21)	5.3 (2.1, 8.6)
	1.0	27% (35)	7.1 (3.8, 10.4)
	2.0	33% (35)	7.0 (3.5, 10.5)

* Mean difference in model-predicted health-related quality of life (HRQOL) for total QOLCE-55 score of patients with and without seizures; higher scores indicate better HRQOL for seizure-free patients.

© 2023 Widjaja E et al. *JAMA Network Open.*

eTable 6. Results of Linear Mixed Model Evaluating a 3-Way Interaction of Time, Surgical Status and Seizure Status

	Estimated difference (95% CI)	p-value
Years follow-up	1.1 (-2.5, 4.6)	.54
Years follow-up squared	-0.3 (-1.9, 1.2)	.68
Surgery (ref = Medical)	-1.0 (-4.7, 2.8)	.61
Seizure-free (ref = Has seizures)	2.3 (-3.3, 7.8)	.42
Age seizure onset	0.6 (0.2, 1.0)	.004
Number of antiseizure medications	-5.5 (-7.3, -3.6)	<.001
Parent, college/university education	1.4 (-2.2, 5.0)	.45
Parent, married or living with spouse	3.8 (-1.0, 8.6)	.12
Family relationships, APGAR	-0.2 (-1.0, 0.6)	.63
Family resources, FIRM	0.6 (0.4.0, 0.8)	<.001
Family demands, FILE	-0.3 (-0.6, 0.1)	.12
Surgery * Years follow-up	5.4 (-1.0, 11.8)	.10
Surgery * Years follow-up squared	-2.0 (-5.0, 1.0)	.19
Seizure-free * Years follow-up	7.3 (-2.5, 17.1)	.14
Seizure-free * Years follow-up squared	-2.5 (-6.3, 1.4)	.21
Surgery * Seizure-free	9.5 (0.6, 18.5)	.037
Surgery * Seizure-free * Years follow-up	-17.5 (-33.0, -1.9)	.028
Surgery * Seizure-free * Years follow-up squared	6.0 (0.0, 11.9)	.049

© 2023 Widjaja E et al. *JAMA Network Open.*

eFigure 2. Model Predicted Health-Related Quality of Life (HRQOL; Total QOLCE-55 Score) Trajectories, Focused on the 3-Way Interaction Between Time, Surgical Status, and Seizure Status

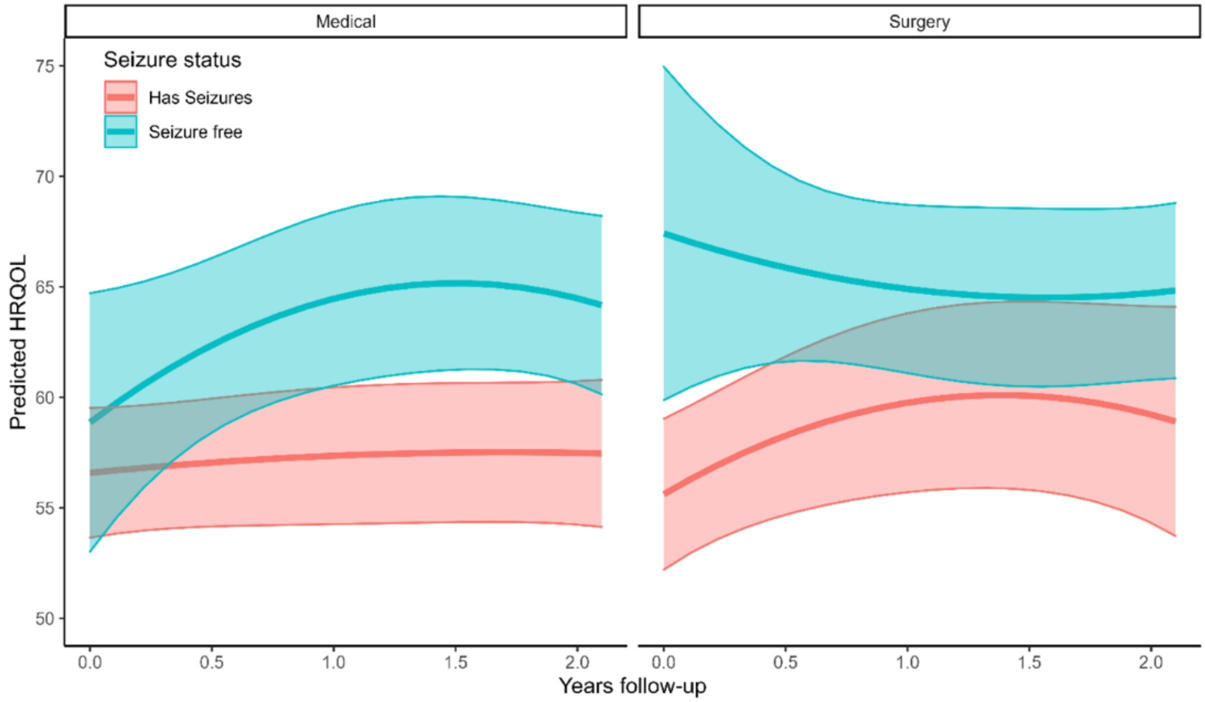

© 2023 Widjaja E et al. *JAMA Network Open.*
