# Supplementary material for: Trajectory of Health-Related Quality of Life After Pediatric Epilepsy Surgery
Source: JAMA Netw Open. 2023 Mar 27;6(3):e234858. doi: 10.1001/jamanetworkopen.2023.4858 (PMC10043749; doi:10.1001/jamanetworkopen.2023.4858)
Supplement: Supplement 2. — Data Sharing Statement [file jamanetwopen-e234858-s002.pdf]

Data Sharing Statement

Widjaja. Trajectory of Health-Related Quality of Life After Pediatric Epilepsy Surgery. *JAMA Netw Open*. Published March 27, 2023. doi:10.1001/jamanetworkopen.2023.4858

Data
Data available: No

Additional Information
Explanation for why data not available: Deidentified data may be shared on reasonable request from qualified investigators, with appropriate approvals from the institutional research ethics boards.
